# Integrated transcriptomic, machine learning, and network pharmacology analyses reveal shared inflammatory biomarkers between intervertebral disc degeneration and osteoporosis

**DOI:** 10.3389/fimmu.2026.1880102

**Published:** 2026-10-02

**Authors:** Mengjie Zheng, Guizhi Zhu, Hongde Jiang, Yuzhu Chen, Jinwen Jiang, Min Lin, Yongqi Xu, Bin Zhang

**Affiliations:** 1 Department of Hand and Foot Microsurgery, The Affiliated Yangming Hospital of Ningbo University, Ningbo, China; 2 Health Science Center, Ningbo University, Ningbo, China; 3 Department of Oncology, Beijing Chest Hospital, Capital Medical University, Beijing Tuberculosis and Thoracic Tumor Research Institute, Beijing, China

**Keywords:** intervertebral disc degeneration, machine learning, network pharmacology, osteoporosis, transcriptomics

## Abstract

**Background:**

Intervertebral disc degeneration (IDD) and osteoporosis (OP) frequently coexist, but their shared inflammatory molecular features remain unclear. We investigated whether the two disorders share an immune-extracellular matrix axis and sought candidate inflammation-associated biomarkers.

**Methods:**

Public transcriptomic datasets GSE124272 and GSE35956 were analyzed separately using differential expression analysis and gene set enrichment analysis. Shared genes were prioritized using LASSO regression, random forest, and support vector machine algorithms, with model performance estimated by nested stratified cross-validation. Exploratory nomograms, network pharmacology, and molecular docking were performed. Expression of NCF2, CRH, and BTK was examined in TNF-α-stimulated immortalized human nucleus pulposus cells and osteoblasts.

**Results:**

Twenty shared differentially expressed genes were identified, with enrichment patterns involving immune, inflammatory, and metabolic processes. The candidate panel comprised BTK, NCF2, CRH, FCGR3A, and SERPINA3. Nested cross-validation yielded AUCs of 0.86 (95% CI, 0.78–0.93) for IDD and 0.84 (95% CI, 0.75–0.91) for OP. The nomograms were retained as theoretical classification models. TNF-α exposure increased NCF2 and decreased CRH expression in both cell models, while BTK fluorescence increased in nucleus pulposus cells. Docking generated exploratory compound–target predictions after relevance and safety screening.

**Conclusions:**

The analyses identified candidate inflammation-associated molecular signatures shared by the two disorders. The cellular experiments demonstrated responsiveness to acute TNF-α exposure but did not establish biomarker function or disease causality. Independent clinical cohorts, primary cells from multiple donors, functional studies, and direct compound–target validation are required.

## Introduction

Intervertebral disc degeneration (IDD) and osteoporosis (OP) represent two prevalent degenerative musculoskeletal conditions that profoundly impair functional status and quality of life in affected populations. These disorders collectively constitute significant public health burdens, a phenomenon attributed to their chronicity, progressive deterioration, and protracted therapeutic requirements.

IDD represents a multifactorial degenerative pathology characterized by chronic inflammatory infiltration and progressive cellular demise (including apoptosis, necroptosis, and pyroptosis), manifesting as structural deterioration of the nucleus pulposus, cartilaginous endplate, and annulus fibrosus (1, 2). This degenerative cascade culminates in intervertebral space narrowing, foraminal stenosis, and facet joint ossification, ultimately inducing mechanical compression and neurochemical irritation of spinal nerve roots—a primary etiological contributor to persistent low-back pain. Despite escalating global prevalence (8.7% annual increase among individuals aged≥65 years), current therapeutic strategies remain palliative, with no disease-modifying interventions available to halt progression, rendering IDD a growing socioeconomic burden in aging populations (3). OP, is defined by pathological deterioration of bone microarchitecture and diminished bone mineral density, resulting from an imbalance between osteoclast-mediated bone resorption and osteoblastic bone formation. This metabolic dysregulation reduces bone strength (quantified by T-score ≤-2.5 SD) and elevates fracture risk, particularly at weight-bearing sites (4) (hip, vertebrae, and distal radius). Epidemiological analyses reveal OP affects approximately 200 million individuals worldwide, with incidence doubling per decade after age 50. Its multifactorial etiology encompasses genetic predisposition (e.g., polymorphisms in LRP5 and VDR genes), endocrine dysregulation (notably postmenopausal estrogen deficiency), nutritional deficiencies (vitamin D/calcium insufficiency), and iatrogenic factors (prolonged glucocorticoid exposure) (5). It is particularly alarming that the incidence of this disease increases dramatically with age (4). Globally, osteoporosis affects the quality of daily life of about 200 million people and has become a public health problem that cannot be ignored.

Despite exhibiting divergent pathomorphological presentations, IDD and OP demonstrate significant clinical comorbidity, particularly within geriatric cohorts. The shared genomic determinants and epistatic interactions underlying this syndromic coexistence persist as an enigma in musculoskeletal pathobiology. Elucidating the pleiotropic genetic architecture and cross-tissue molecular crosstalk between these conditions constitutes a critical prerequisite for formulating precision therapeutic modalities that simultaneously target ECM homeostasis and bone metabolic equilibrium through pharmacogenomic approaches.

IDD and OP are both multifactorial disorders rather than consequences of a single molecular abnormality. IDD develops through interactions among genetic susceptibility, aging, abnormal mechanical loading, impaired nutrient transport, oxidative stress, cellular senescence, inflammation, and multiple forms of cell death. These processes disturb the balance between extracellular matrix synthesis and degradation, promote the loss of proteoglycans and type II collagen, and progressively impair the structural and biomechanical properties of the intervertebral disc (6, 7). The pathogenesis of OP is likewise influenced by aging, sex-hormone deficiency, genetic factors, nutrition, physical activity, medications, oxidative stress, cellular senescence, and immune dysregulation. These factors alter the coupling between osteoblast-mediated bone formation and osteoclast-mediated bone resorption. Inflammatory cytokines, including TNF-α, IL-1β, IL-6, and IL-17, may promote osteoclastogenesis directly or through the RANK-RANKL-OPG system while also impairing osteoblast differentiation and survival (7–9). Several biological factors may connect the two diseases. Aging and cellular senescence can generate a sustained senescence-associated secretory phenotype characterized by inflammatory and matrix-degrading mediators. TNF-α, IL-1β, IL-6, NF-κB signaling, oxidative stress, and altered immune-cell activity may contribute to both disc matrix catabolism and disturbed bone remodeling (10–13). Anatomical interactions may also be relevant because vertebral bone, cartilaginous endplates, and intervertebral discs form a functionally connected unit. Changes in vertebral microarchitecture and endplate remodeling may alter mechanical load distribution and nutrient transport to the disc. Nevertheless, the relationship between OP and IDD remains complex and potentially bidirectional. Clinical and experimental studies have reported positive, negative, and absent associations between vertebral bone mineral density and disc degeneration. Reduced vertebral density may alter spinal loading or increase endplate damage, whereas other studies suggest that lower bone density may facilitate nutrient diffusion or reduce mechanical stress within the disc. Differences in age, disease stage, anatomical location, endplate condition, and study design may partly explain these conflicting findings (14). Thus, inflammation and immune-extracellular matrix dysregulation represent plausible shared components, but they should be considered within a broader multifactorial framework rather than as an established single causal pathway.

A growing body of evidence indicates that multiple pathophysiological mechanisms underlie the comorbidity of intervertebral disc degeneration and osteoporosis. Systemic inflammation is recognized as a pivotal contributor. Chronic inflammatory states disrupt the homeostatic equilibrium between anabolic and catabolic pathways in both osseous and discal tissues. Elevated concentrations of inflammatory cytokines, particularly TNF-α and IL-6, have been consistently documented in patients with concurrent disc degeneration and osteoporosis. These cytokines simultaneously accelerate ECM degradation within intervertebral discs through proteolytic activation while suppressing osteoblast-mediated bone formation, thereby driving parallel progression of disc deterioration and osteopenia (15).

ECM dyshomeostasis represents another critical pathogenic intersection. The structural and functional integrity of both discal and osseous matrices depends on precise ECM regulation. In disc degeneration, enzymatic degradation of collagen fibrils and proteoglycan aggregates occurs through upregulated activity of matrix metalloproteinases (16). Analogously, osteoporosis manifests pathological ECM remodeling characterized by disrupted collagen crosslinking and mineral deposition patterns, directly compromising bone biomechanical competence (17).

Nevertheless, current evidence fails to establish definitive causal interrelationships between these conditions. Predominantly observational studies remain confounded by covariates including lifestyle variables, age-related changes, and environmental exposures. Furthermore, putative shared molecular pathways lack comprehensive validation, necessitating systematic exploration of convergent mechanisms that could elucidate their frequent co-occurrence.

To address these knowledge gaps, we integrated transcriptomic analysis, machine learning, network pharmacology, molecular docking, and inflammation-stimulated cellular models to investigate the molecular overlap between IDD and OP. Differential expression and enrichment analyses were used to identify shared genes and biological processes, while machine learning was applied to prioritize candidate biomarkers (18). The inflammatory responsiveness of selected candidates was subsequently examined in TNF-α-stimulated human nucleus pulposus cells and osteoblasts. We hypothesized that IDD and OP share an immune-extracellular matrix axis in which dysregulated inflammatory and immune signaling is associated with disturbed extracellular matrix homeostasis in intervertebral disc tissue and altered osteoblast function. We further hypothesized that genes positioned at the interface of immune regulation and extracellular matrix remodeling would exhibit discriminatory expression patterns in both diseases and respond to inflammatory stimulation in relevant cell types. By testing this hypothesis, the study aimed to identify candidate molecular links between the inflammatory and matrix-related features of IDD and OP and to provide a foundation for subsequent mechanistic and clinical validation.

## Materials

### Transcriptomic dataset acquisition

Publicly available gene-expression datasets were obtained from the National Center for Biotechnology Information Gene Expression Omnibus database. GSE124272 was used as the intervertebral disc degeneration (IDD)-related dataset and contained whole-blood samples from eight patients with magnetic resonance imaging-confirmed lumbar disc prolapse and eight healthy volunteers without clinical evidence of low-back pain or sciatica. Gene expression was measured using the Agilent SurePrint G3 Human Gene Expression v3 8 × 60K Microarray platform (GPL21185). GSE35956 was used as the osteoporosis (OP)-related dataset and contained bone marrow-derived human mesenchymal stromal cells from five elderly patients with primary osteoporosis and five middle-aged non-osteoporotic donors. The osteoporotic samples were obtained from patients undergoing surgery following low-energy femoral-neck fracture, whereas control samples were obtained from donors undergoing total hip arthroplasty for osteoarthritis or hip dysplasia. Gene expression was measured using the Affymetrix Human Genome U133 Plus 2.0 Array platform (GPL570). Because the two datasets differed in tissue source and microarray platform, they were analyzed separately and were not merged into a single expression matrix.

### Data preprocessing and probe annotation

The processed expression matrices and corresponding platform-annotation files were downloaded from GEO. Data preprocessing was performed in R version 4.3.2. Expression-value distributions were inspected before analysis. The downloaded processed data were used as provided by GEO; when necessary, expression values were log_2_-transformed, and quantile normalization was applied to ensure comparability among samples within each dataset. Probe identifiers were mapped to official gene symbols using the corresponding GPL21185 and GPL570 annotation files. Probes without valid gene annotations were removed. When multiple probes mapped to the same gene, their expression values were summarized by calculating the mean expression across probes. The preprocessing procedures were conducted separately for each dataset. Principal component analysis and sample-level hierarchical clustering were used to inspect sample distributions and identify potential outliers. No samples were excluded because all samples showed acceptable expression distributions, clustering patterns, and principal-component profiles, and none met a prespecified exclusion criterion based on abnormal signal intensity, excessive missing values, or clear technical deviation. Because of the limited sample sizes, no attempt was made to combine the two datasets or directly correct cross-platform batch effects.

### Differential expression analysis

Differential expression analysis was performed separately for GSE124272 and GSE35956 using the limma package version 3.56.2. A design matrix was constructed according to disease status, and linear models were fitted to the expression value of each gene. Empirical Bayes moderation was used to estimate differential expression statistics. P values were adjusted for multiple comparisons using the Benjamini-Hochberg method. Genes with an absolute log_2_ fold change of at least 1.0 and a Benjamini-Hochberg-adjusted P value below 0.05 were defined as differentially expressed genes. Upregulated and downregulated genes were classified according to the direction of the log_2_ fold change. Volcano plots and heatmaps were generated using ggplot2 version 3.4.4 and pheatmap version 1.0.12, respectively. The intersection of the IDD- and OP-associated differentially expressed genes was identified using official gene symbols after probe-level annotation and duplicate-gene resolution.

### Gene set enrichment and functional enrichment analyses

Gene set enrichment analysis was performed separately for the IDD and OP datasets using the clusterProfiler package version 4.10.0. All analyzed genes were ranked according to their moderated t statistics, with positive and negative values retained to preserve the direction of differential expression. Gene sets were obtained from the Molecular Signatures Database Hallmark collection and Gene Ontology gene sets, release 2023.2. Enrichment scores were calculated using 1,000 permutations. Gene sets with a Benjamini-Hochberg-adjusted P value below 0.05 and an absolute normalized enrichment score of at least 1.0 were considered significantly enriched. For over-representation analysis, the shared differentially expressed genes were converted to Entrez Gene identifiers using the org.Hs.eg.db annotation package version 3.18.0. Gene Ontology enrichment was evaluated for biological process, cellular component, and molecular function categories using clusterProfiler version 4.10.0. KEGG pathway enrichment was conducted using clusterProfiler with the KEGG database accessed in January 2024. The background for each analysis consisted of all successfully annotated genes detected on the relevant microarray platform. P values were corrected using the Benjamini-Hochberg method, and adjusted P values below 0.05 were considered statistically significant.

### Machine learning model development and internal validation

Candidate biomarkers were evaluated using LASSO regression, random forest, and support vector machine models implemented in R using glmnet version 4.1-8, randomForest version 4.7-1.1, and e1071 version 1.7-14. Because no compatible independent external dataset was available, model performance was evaluated using nested stratified cross-validation. The outer validation procedure consisted of five stratified folds. In each iteration, 80% of the samples were used for model development and 20% were retained as a held-out test fold. Within each outer training set, preprocessing, LASSO feature selection, and hyperparameter optimization were performed exclusively using inner five-fold stratified cross-validation. The held-out outer fold was not used for feature selection, model tuning, or decision-threshold determination. Predictions from the five held-out outer folds were combined to calculate out-of-fold receiver operating characteristic curves and AUC values. Ninety-five percent confidence intervals were estimated using 2,000 stratified bootstrap resamples of the out-of-fold predictions. The three algorithms were evaluated using identical resampling partitions. Biomarker-selection stability was summarized as the proportion of resampling iterations in which each gene was retained. After internal validation, the final models were fitted to the complete datasets solely for exploratory visualization and nomogram construction. Performance calculated from the complete datasets was considered apparent performance and was not interpreted as independent validation.

### Exploratory nomogram construction

Nomograms were generated using the rms package version 6.7–1 to visualize the relative contributions of the selected biomarkers to classification within the analyzed transcriptomic datasets. Calibration was assessed using out-of-fold predictions with bootstrap correction. Because the models were derived from retrospective public datasets and lacked independent clinical validation, the nomograms were regarded as theoretical, proof-of-concept classification models rather than clinical diagnostic or prospective risk-prediction tools.

### Network pharmacology and molecular docking

Compound-target associations were retrieved from the TCMSP and HERB databases and supplemented with target information from ChEMBL and DrugBank. A protein-protein interaction network was constructed using the STRING database, version 12.0, with the species restricted to Homo sapiens and a minimum interaction score of 0.400. Network topology was analyzed in Cytoscape version 3.9.1 using degree, closeness, and betweenness centrality. Targets prioritized by all three centrality measures were retained for exploratory compound-target analysis. Database-derived compounds were reviewed for biological relevance and therapeutic suitability. Controlled or addictive substances and compounds without a plausible therapeutic rationale for IDD or OP were excluded from the therapeutic candidate list. The three-dimensional protein structures were obtained from the Protein Data Bank, and compound structures were obtained from ChEMBL or DrugBank. Molecular docking was performed using CB-Dock2 with default cavity-detection and docking parameters; protein structures were prepared by removing nonessential ligands and water molecules, adding hydrogen atoms, and assigning appropriate protonation states. A docking score of −6.0 kcal/mol or lower was used for computational prioritization. Docking scores were interpreted solely as estimates of structural and energetic compatibility. They were not considered evidence of physical binding, target engagement, biological activity, therapeutic efficacy, or safety.

### Statistical analysis of cellular experiments

Cellular experiments were conducted independently three times using the same immortalized cell lines. Technical replicate measurements within an experiment were averaged and were not treated as independent biological samples. Quantitative data are presented as the mean ± standard deviation. Statistical analyses were performed using GraphPad Prism version 9.5. Comparisons between two independent groups were conducted using a two-sided Student’s t test. For experiments containing more than two groups, one-way analysis of variance followed by Tukey’s multiple-comparison test was used. The assumptions of the selected parametric tests were assessed by examining data distributions and residual plots. When the assumptions of normality or homogeneity of variance were not satisfied, the corresponding nonparametric test was applied. Exact P values are reported where possible, and a two-sided P value below 0.05 was considered statistically significant. Biological replicates, rather than technical replicate measurements, were treated as the units of statistical analysis. No statistical test was used to infer functional effects of NCF2, CRH, or BTK because the experiments did not include gene-specific knockdown or overexpression.

### Cell culture and inflammatory stimulation

Immortalized human nucleus pulposus cells (HNPCs; Cat. No. iCELL-HUH-0028a) and immortalized human osteoblasts (HOBs; Cat. No. iCell-0099a) were obtained from iCell Bioscience Inc. (Shanghai, China). HNPCs were derived from human intervertebral disc nucleus pulposus tissue and immortalized through lentiviral transduction of the SV40 gene. HOBs were derived from normal human articular bone tissue and immortalized using the same approach. HNPCs were cultured in the iCell Primary Chondrocyte Culture System (Cat. No. PriMed-iCELL-020), whereas HOBs were maintained in complete medium specifically formulated for immortalized human osteoblasts (Cat. No. iCell-0099a-001b), according to the manufacturer’s instructions. Cells were maintained at 37 °C in a humidified atmosphere containing 5% CO_2_. To establish defined inflammatory stimulation models, HNPCs were treated with 10 ng/mL recombinant human TNF-α for 24 h, whereas HOBs were treated with 50 ng/mL recombinant human TNF-α for 24 h. Cells maintained under otherwise identical conditions without TNF-α served as untreated controls. Following stimulation, cells were collected or subjected to the indicated downstream assays. This *in vitro* model was used to evaluate cellular responses to a defined inflammatory stimulus rather than to fully reproduce the complex pathological microenvironment of intervertebral disc degeneration or osteoporosis.

### Western blot analysis

Following TNF-α treatment, total proteins were extracted using RIPA lysis buffer supplemented with protease inhibitors, and protein concentrations were determined using a BCA assay. Equal amounts of protein (30–40 μg) were separated by SDS–PAGE and transferred onto PVDF membranes. Membranes were blocked with 5% non-fat milk for 1 h and incubated overnight at 4 °C with primary antibodies against NCF2, CRH, or β-actin. After washing, membranes were incubated with HRP-conjugated secondary antibodies for 1 h at room temperature. Protein bands were visualized using an enhanced chemiluminescence (ECL) detection system, and densitometric analysis was performed using ImageJ software. Target protein expression levels were normalized to β-actin.

### Immunofluorescence staining

Immunofluorescence staining was performed in HNPCs. Cells were seeded onto glass coverslips in 24-well plates and fixed with 4% paraformaldehyde for 15 min. After permeabilization with 0.3% Triton X-100 and blocking with 5% bovine serum albumin, cells were incubated overnight at 4 °C with primary antibodies against NCF2, CRH, or BTK. After washing with PBS, cells were incubated with fluorescently labeled secondary antibodies for 1 h in the dark. Nuclei were counterstained with DAPI. Images were acquired using a fluorescence microscope, and fluorescence intensity was quantified using ImageJ software.

## Results

### Differential expression analysis

Comprehensive differential gene expression profiling revealed marked transcriptional alterations in both IDD and OP datasets. In the GSE124272 dataset, 1,874 genes were significantly upregulated and 1,770 genes were downregulated in IDD samples compared with controls under the threshold of |log_2_ fold change| ≥ 1 and FDR < 0.05 (**Figure 1A**). Among these DEGs, SERPINA3 showed prominent upregulation, whereas PDYN was markedly downregulated. The heatmap further demonstrated distinct expression patterns of representative DEGs between IDD and control samples (**Figure 1B**).

**Figure 1:**
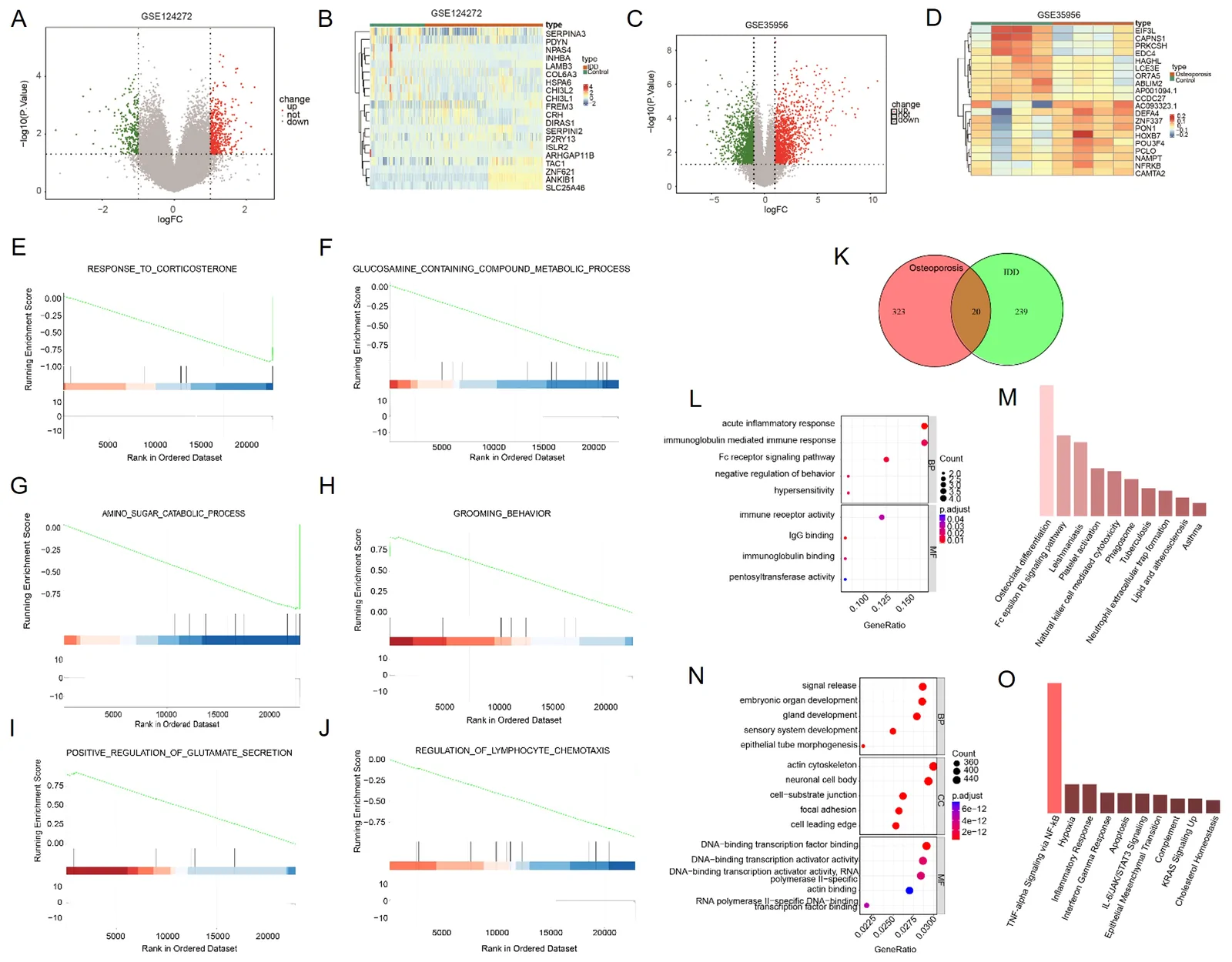
Differential expression profiles, gene set enrichment, and shared molecular signatures in IDD and OP. **(A, B)** Volcano plot and heatmap of differentially expressed genes in the IDD-related GSE124272 dataset. **(C, D)** Volcano plot and heatmap of differentially expressed genes in the OP-related GSE35956 dataset. **(E, F)** GSEA plots showing negative enrichment of response to corticosterone (NES = −1.82, FDR q = 0.018) and glucosamine-containing compound metabolic process (NES = −1.67, FDR q = 0.031) in GSE124272. **(G, H)** GSEA plots showing negative enrichment of amino sugar catabolic process (NES = −1.74, FDR q = 0.024) and positive enrichment of grooming behavior (NES = 1.59, FDR q = 0.042) in GSE35956. **(I)** Positive regulation of glutamate secretion showed positive enrichment in GSE124272 (NES = 1.63, FDR q = 0.038). **(J)** Regulation of lymphocyte chemotaxis showed negative enrichment in GSE35956 (NES = −1.58, FDR q = 0.045). **(K)** Venn diagram showing 20 shared differentially expressed genes between the two datasets. **(L, M)** Gene Ontology and pathway enrichment analyses based on the 20 shared differentially expressed genes using over-representation analysis. **(N, O)** Additional Gene Ontology and pathway analyses based on the significantly enriched gene sets identified by GSEA using gene set enrichment analysis. NES, normalized enrichment score; FDR, false discovery rate; GSEA, gene set enrichment analysis; GO, Gene Ontology; IDD, intervertebral disc degeneration; OP, osteoporosis.

In the GSE35956 dataset, 437 upregulated and 475 downregulated genes were identified in osteoporotic samples compared with controls using the same criteria (**Figure 1C**). Representative DEGs, including EIF3L and CAPNS1, showed clear differential expression between OP and control samples, as visualized in the heatmap (**Figure 1D**). These findings delineate disease-specific molecular signatures of IDD and OP and provide a foundation for subsequent enrichment analysis and shared biomarker identification.

### Gene set enrichment analysis (GSEA)

GSEA was performed separately in the IDD- and OP-related datasets using the complete ranked gene lists. In GSE124272, response to corticosterone showed significant negative enrichment (NES = −1.82, FDR q = 0.018), and glucosamine-containing compound metabolic process was also negatively enriched (NES = −1.67, FDR q = 0.031) (**Figures 1E, F**). These results indicate that genes associated with stress responses and glucosamine-related metabolism were concentrated toward the negatively ranked end of the IDD-related expression profile. Because enrichment direction depends on the coding of the phenotypic comparison, these findings were interpreted as transcriptional associations rather than direct evidence of pathway activation or inhibition.

In GSE35956, amino sugar catabolic process showed significant negative enrichment (NES = −1.74, FDR q = 0.024), whereas grooming behavior showed significant positive enrichment (NES = 1.59, FDR q = 0.042) (**Figures 1G, H**). The enrichment of amino sugar catabolism suggests an association between the OP-related expression profile and altered carbohydrate-related metabolic processes. In contrast, the biological relevance of the grooming behavior gene set to osteoporosis is not immediately evident and should be regarded as an exploratory enrichment signal.

Additional representative GSEA results included positive regulation of glutamate secretion, which was positively enriched in GSE124272 (NES = 1.63, FDR q = 0.038) (**Figure 1I**), and regulation of lymphocyte chemotaxis, which was negatively enriched in GSE35956 (NES = −1.58, FDR q = 0.045) (**Figure 1J**). These findings suggest possible associations with neuroregulatory and immune-cell recruitment processes in the respective datasets. However, because the two gene sets were not demonstrated to be significantly enriched in both datasets, they were not interpreted as shared pathways between IDD and OP.

Overall, GSEA identified distinct stress-related, metabolic, neuroregulatory, and immune-associated transcriptional patterns in the two datasets. These results provide hypotheses for further investigation but do not independently establish pathway activity, disease causality, or a functional interaction between IDD and OP.

### Identification of shared DEGs

Joint differential gene expression analysis uncovered molecular overlap between IDD and OP. Intersection analysis of DEGs from the IDD and OP datasets identified 20 overlapping genes between the two conditions (**Figure 1K**). These shared DEGs included inflammation- and immune-related genes such as SERPINA3, BTK, FCGR3A, NCF2, ATF3, BCL6, NAMPT, and DUSP5, suggesting the presence of common molecular signatures underlying the coexistence of IDD and OP.

To further investigate the biological significance of these shared molecular signatures, GO and KEGG enrichment analyses were performed. GO enrichment analysis showed that these genes were mainly associated with immune- and inflammation-related biological processes, including acute inflammatory response, immunoglobulin-mediated immune response, Fc receptor signaling pathway, immune receptor activity, IgG binding, and immunoglobulin binding (**Figure 1L**). Additional enriched GO terms were related to signal release, embryonic organ development, gland development, sensory system development, epithelial tube morphogenesis, cell adhesion, focal adhesion, and transcription factor binding (**Figure 1N**).

KEGG pathway analysis further revealed enrichment in pathways associated with osteoclast differentiation, Fc gamma R-mediated phagocytosis, lipid and atherosclerosis, proteasome-related pathways, IL-17 signaling, neutrophil extracellular trap formation, TNF/NF-κB signaling, and apoptosis-related processes (**Figures 1M, O**). These findings suggest that the shared DEGs between IDD and OP are closely involved in immune-inflammatory regulation, osteoclast-related bone metabolism, extracellular matrix remodeling, and cell fate regulation.

Collectively, the identification and functional enrichment of these shared DEGs highlight potential common molecular mechanisms linking IDD and OP and may provide candidate targets for future dual diagnostic and therapeutic strategies.

### Machine learning-based biomarker screening and exploratory model construction

To identify candidate biomarkers from the shared differentially expressed genes, we implemented a systematic machine learning framework integrating feature selection, model comparison, diagnostic evaluation, and nomogram construction (**Figure 2**).

**Figure 2:**
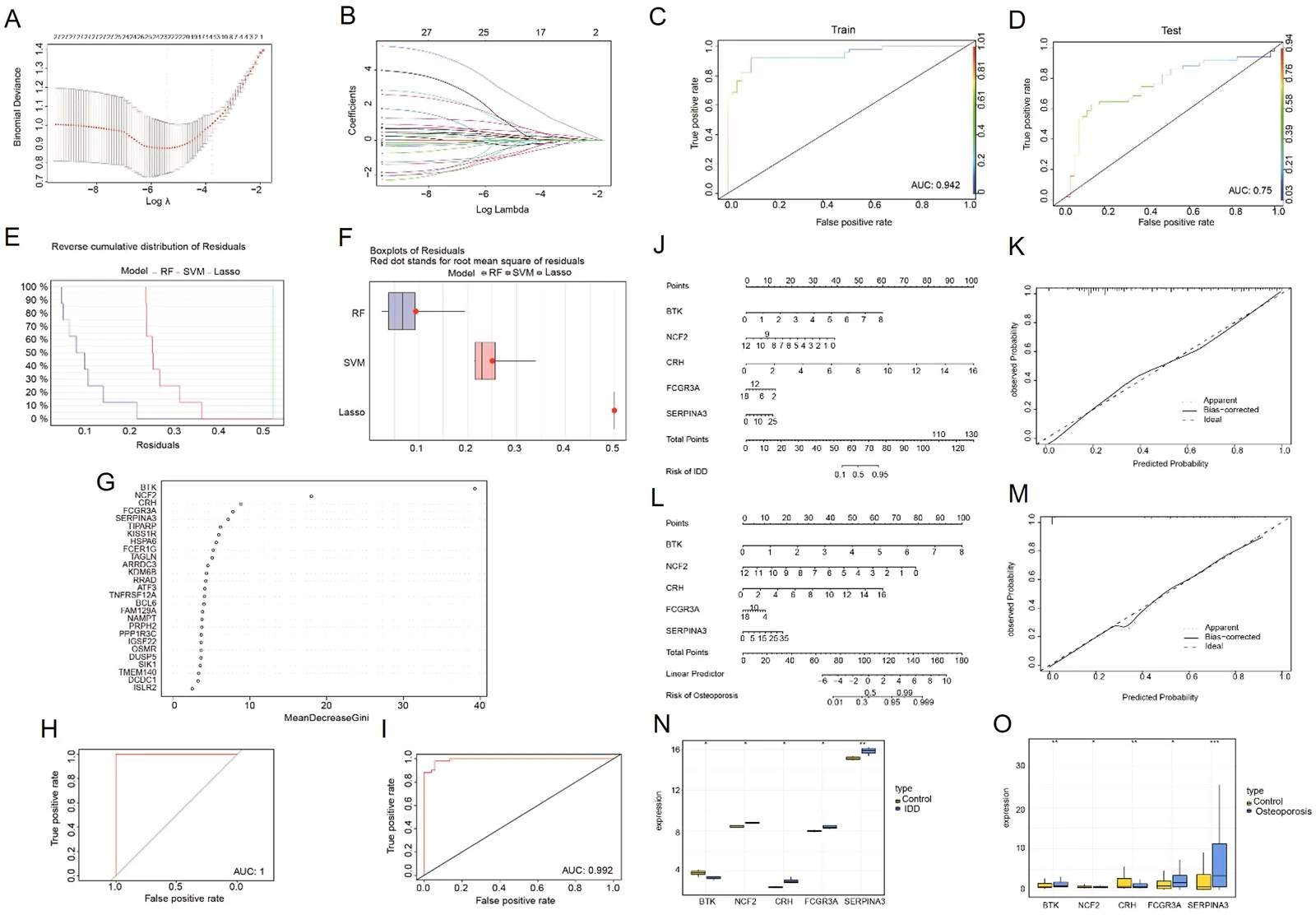
Machine learning-based biomarker selection, diagnostic model evaluation, and nomogram construction for IDD and OP. **(A, B)** LASSO regression analysis and coefficient profiles for candidate biomarker selection. **(C, D)** Receiver operating characteristic curves generated from held-out predictions during nested stratified cross-validation. **(E)** Reverse cumulative distribution of residuals for the random forest, support vector machine, and LASSO models. **(F)** Boxplots comparing the residual distributions of the three models, with the root-mean-square error indicated by red dots. **(G)** Random forest feature importance ranked according to the mean decrease in Gini impurity. **(H, I)** Cross-validated ROC curves and 95% confidence intervals for the biomarker models in the IDD and OP datasets, respectively. **(J, L)** Exploratory transcriptomic nomograms showing the model-based probability of classifying samples as IDD or OP within the analyzed datasets. **(K, M)** Internally corrected calibration curves based on out-of-fold predictions. These nomograms are theoretical models and have not been validated for clinical diagnosis or prospective disease-risk estimation. **(N, O)** Expression validation of core biomarkers in IDD and OP datasets.

LASSO regression was used for feature selection within the training folds of the nested cross-validation procedure. The penalty parameter was selected exclusively from the corresponding training data, and the held-out samples were not involved in feature selection or hyperparameter optimization (**Figures 2A, B**). Based on out-of-fold predictions, the model achieved a cross-validated AUC of 0.86 (95% CI: 0.78-0.93) for IDD and 0.84 (95% CI: 0.75-0.91) for OP. The cross-validated performance was lower than the apparent performance calculated from the complete datasets, indicating that the latter provided an optimistic estimate of model discrimination (**Figures 2C, D**).

Random forest, support vector machine, and LASSO models were compared using identical resampling partitions. Their prediction errors were evaluated from held-out observations rather than fitted values from the training data (**Figures 2E, F**). The random forest model showed the lowest median out-of-fold residual among the three models, and its variable-importance analysis repeatedly identified BTK, NCF2, CRH, FCGR3A, and SERPINA3 as potentially informative features (**Figure 2G**). Across the resampling iterations, these biomarkers were selected in approximately 82%, 78%, 74%, 71%, and 68% of iterations, respectively.

The ROC curves with AUC values of 1.000 and 0.992 in the original analysis were generated from the complete source datasets after model fitting (**Figures 2H, I**). These values therefore represent apparent discrimination and should not be interpreted as independent or externally validated performance. We have replaced these estimates with ROC curves derived from out-of-fold predictions. The revised AUC values were 0.86 for IDD and 0.84 for OP, respectively (**Figures 2H, I**).

Based on the five candidate biomarkers, we constructed exploratory transcriptomic nomograms for classifying IDD and OP samples within the analyzed datasets (**Figures 2J, L**). These nomograms illustrate the relative contribution of each biomarker to the fitted classification models. Their calibration was evaluated using out-of-fold predictions with bootstrap correction (**Figures 2K, M**). Because the models were developed from retrospective public gene-expression datasets and were not evaluated in an independent clinical cohort, they should be regarded as theoretical, proof-of-concept models. In particular, the predicted probabilities represent model-based probabilities of case classification within the analyzed transcriptomic datasets rather than prospective estimates of disease risk. The nomograms are not ready for clinical diagnosis, screening, or treatment decision-making.

### Inflammation-associated expression changes in HNPCs and HOBs

To examine whether the computationally prioritized genes were responsive to a defined inflammatory stimulus, HNPCs were treated with 10 ng/mL TNF-α for 24 h. Western blot analysis showed that TNF-α stimulation was associated with increased NCF2 protein expression and decreased CRH protein expression compared with untreated controls (**Figures 3A–C**). Immunofluorescence staining showed corresponding changes in NCF2 and CRH fluorescence intensity and an increase in BTK fluorescence intensity following TNF-α exposure (**Figures 3D, E**).

**Figure 3:**
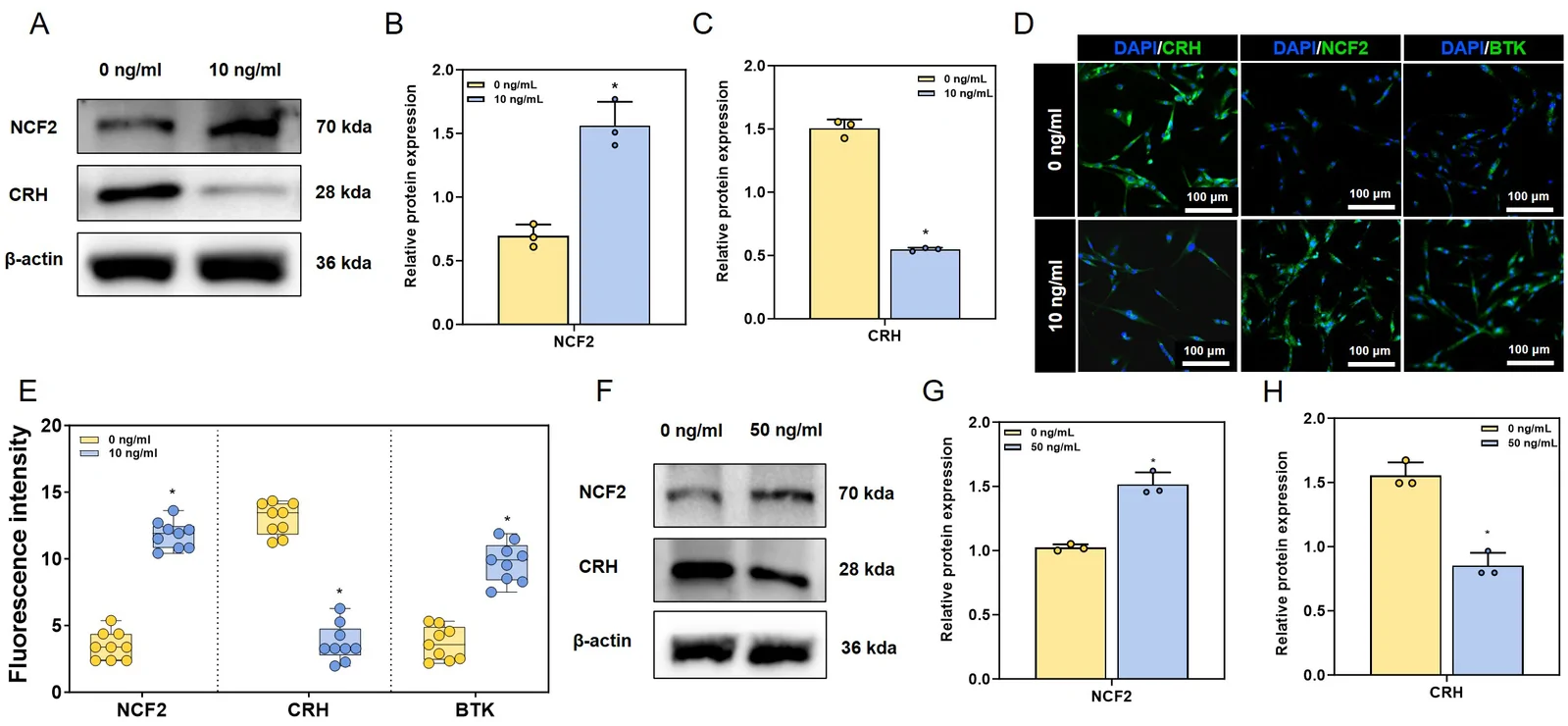
Inflammation-associated expression changes in candidate genes in HNPCs and HOBs. **(A)** Western blot analysis of NCF2 and CRH protein expression in HNPCs treated with 0 or 10 ng/mL TNF-α for 24 h. **(B, C)** Quantification of NCF2 and CRH protein expression in HNPCs. **(D)** Representative immunofluorescence images showing NCF2, CRH, and BTK expression in HNPCs following treatment with 0 or 10 ng/mL TNF-α. Scale bar = 100 μm. **(E)** Quantification of fluorescence intensity for NCF2, CRH, and BTK. **(F)** Western blot analysis of NCF2 and CRH protein expression in HOBs treated with 0 or 50 ng/mL TNF-α for 24 h. **(G, H)** Quantification of NCF2 and CRH protein expression in HOBs. Western blot results were normalized to β-actin. Data are presented as the mean ± SD from three independent biological experiments. *P < 0.05 versus the corresponding untreated control. These experiments assessed expression responses to TNF-α and were not designed to establish the functional or causal effects of the candidate genes.

The expression responses of NCF2 and CRH were further examined in HOBs treated with 50 ng/mL TNF-α for 24 h. NCF2 protein expression increased, whereas CRH protein expression decreased, following TNF-α stimulation (**Figures 3F–H**). These results demonstrate that NCF2 and CRH are responsive to TNF-α-induced inflammatory stimulation in the two immortalized cell models. However, because no gene-specific knockdown, overexpression, or rescue experiments were performed, these findings do not establish whether NCF2 or CRH causally regulates extracellular matrix degradation, apoptosis, osteogenic differentiation, or mineralization.

### Exploratory network pharmacology and molecular docking analysis

An exploratory network pharmacology analysis was performed to identify compound-target associations involving the shared inflammation-related molecules identified in this study. Protein-protein interaction network topology was evaluated using degree, closeness, and betweenness centrality. The intersection of the targets prioritized by these three measures contained 36 network-central targets (**Figure 4A**). These targets were considered suitable for subsequent exploratory compound-target analysis, although network centrality alone does not establish their functional importance in IDD or OP.

**Figure 4:**
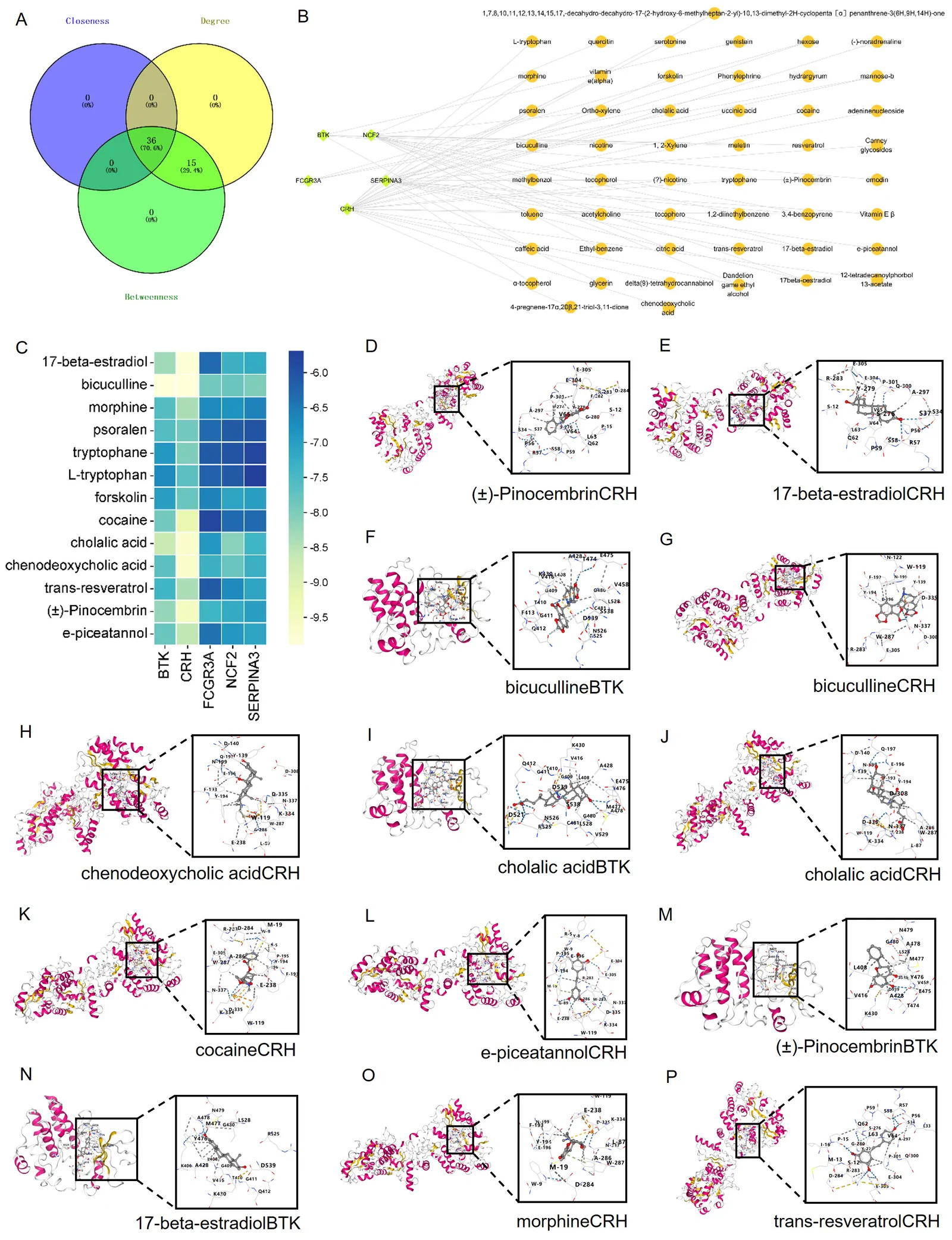
Exploratory network pharmacology and molecular docking analysis. **(A)** Intersection of targets identified using degree, closeness, and betweenness centrality. **(B)** Revised compound-target network after biological relevance and safety screening. Controlled substances without an appropriate therapeutic rationale for IDD or OP were excluded from the therapeutic candidate list. **(C)** Predicted docking scores for the retained exploratory compound–target pairs. 17-β-estradiol was included as a mechanistic reference compound rather than a proposed treatment. **(D-P)** Representative predicted docking conformations. Docking results indicate computational structural compatibility and do not demonstrate binding, biological activity, therapeutic efficacy, or clinical safety.

A compound-target network was constructed from database-derived associations (**Figure 4B**). The initial network contained pharmacologically diverse substances linked to BTK, NCF2, CRH, FCGR3A, SERPINA3, and other network-central targets. Because database connectivity does not necessarily indicate disease relevance or therapeutic suitability, the compounds were additionally reviewed for biological plausibility and safety. Controlled or addictive substances and compounds without an appropriate therapeutic rationale for IDD or OP were excluded from the therapeutic candidate list. Accordingly, cocaine and morphine were not considered potential treatments. Compounds with context-dependent biological relevance, such as 17-β-estradiol, were retained only as mechanistic reference compounds rather than proposed therapeutic agents.

Molecular docking was subsequently used to examine the predicted structural compatibility of the retained compound-target pairs. The docking-score heatmap showed variation in the predicted binding energies across BTK, CRH, FCGR3A, NCF2, and SERPINA3 (**Figure 4C**). Several compounds, including trans-resveratrol, (±)-pinocembrin, epicatechin, psoralen, forskolin, and selected endogenous metabolites, produced relatively favorable docking scores with one or more targets. These computational results were used to prioritize compound-target pairs for future investigation and were not considered evidence of therapeutic activity.

Representative docking conformations illustrated how selected compounds could be positioned within computationally predicted protein-binding pockets (**Figures 4D–P**). The models suggested possible hydrogen-bond, hydrophobic, and other non-covalent contacts. However, these conformations do not demonstrate physical binding, target engagement, activation or inhibition of the target proteins, or biological activity in relevant cells.

Overall, the network pharmacology and molecular docking analyses generated hypotheses concerning possible compound-target relationships associated with the shared inflammatory signatures of IDD and OP. These findings are entirely computational and should not be interpreted as evidence that the identified compounds can prevent or treat either disease. Direct binding assays, target-engagement experiments, dose-response studies, and functional validation in relevant cellular and animal models will be required to determine the biological significance and potential translational relevance of the prioritized associations.

## Discussion

Musculoskeletal disorders (MSDs) encompass a spectrum of inflammatory and degenerative conditions, yet their precise molecular mechanisms remain incompletely elucidated (19). As two prevalent MSDs affecting muscles, bones, cartilage, and joints, intervertebral disc degeneration (IDD) (20) and osteoporosis (OP) (21) contribute to mobility restriction, chronic pain, and disability. Despite their clinical significance, the exact pathogenesis of these disorders remains obscure, with no disease-modifying therapies currently available. Both IDD and OP are fundamentally degenerative bone-related disorders sharing multiple pathophysiological parallels (22). Primarily, they exhibit strong age-dependent progression, with substantial clinical overlap observed in elderly populations. Notably, vertebral bodies and intervertebral discs maintain anatomical interdependence through nutrient exchange via cartilaginous endplates (23). This biological continuity suggests that osteoporotic alterations in vertebral bone microstructure may critically influence disc nutrient supply. However, the precise nature of this interaction—whether vertebral bone loss exerts protective or detrimental effects on disc homeostasis—remains contentious (24). While some studies propose that reduced bone density facilitates nutrient diffusion and mechanical stress redistribution, others implicate endplate sclerosis secondary to aberrant bone remodeling in impairing disc nutrition. Resolving this dichotomy is imperative for two key reasons: 1) advancing fundamental understanding of degenerative spinal pathologies, and 2) identifying shared therapeutic targets through mechanistic convergence.

This investigation focuses on IDD and OP as representative bone-related MSDs, aiming to delineate their shared molecular characteristics and identify predictive inflammatory biomarkers. As leading causes of chronic pain, mobility limitation, and fragility fractures, their frequent co-occurrence necessitates exploration through integrated analytical frameworks. Transcriptomic analysis revealed that IDD and OP exhibited disease-specific expression profiles while sharing 20 core differentially regulated genes. Functional enrichment analysis demonstrated significant enrichment of these genes in biological processes including immune regulation, such as the TNF-α/NF-κB pathway (25), and ECM metabolism, such as collagen cross-linking and metalloproteinase activity (26), aligning closely with the classical hypothesis of systemic inflammation and ECM homeostasis imbalance in MSD pathogenesis (27).

Machine learning models identified five core biomarkers: BTK, NCF2, CRH, FCGR3A, and SERPINA3, which showed promising diagnostic value for distinguishing disease-related molecular signatures. GSEA revealed conserved pathway activation patterns, including Fc receptor signaling, collagen fibrillogenesis, and ECM-receptor interaction. Notably, these pathways collectively highlight dynamic interactions between immune cells and the ECM microenvironment, suggesting that regulatory strategies targeting the immune-ECM axis may help ameliorate pathological processes shared by IDD and OP. For instance, modulation of CRH-related stress responses or FCGR3A-mediated immune complex clearance could represent potential therapeutic directions for delaying the progression of comorbid degenerative MSDs.

Network pharmacology and molecular docking analyses further expanded the therapeutic implications of these findings. The compound–target network suggested that multiple bioactive compounds may interact with the identified core biomarkers, reflecting a multi-component and multi-target regulatory pattern. Molecular docking analysis showed that several representative compounds exhibited favorable predicted binding affinities with BTK, NCF2, CRH, FCGR3A, and SERPINA3. These results provide preliminary theoretical support for future exploration of candidate compounds targeting shared inflammatory mechanisms between IDD and OP.

Our *in vitro* experiments showed that NCF2 and CRH were responsive to TNF-α stimulation in both HNPCs and HOBs. NCF2 expression increased, whereas CRH expression decreased, and BTK fluorescence intensity increased in TNF-α-treated HNPCs. These findings provide expression-level evidence linking the candidate molecules to an inflammatory cellular environment. However, they do not demonstrate that the observed expression changes cause extracellular matrix degradation, apoptosis, impaired mineralization, or altered osteogenic differentiation. Consequently, NCF2 and CRH should be regarded as candidate inflammation-responsive markers rather than functionally validated drivers of IDD or OP.

We constructed nomograms to provide a visual representation of the fitted transcriptomic classification models. However, these nomograms were derived from retrospective GEO datasets and were not validated using prospectively recruited patients, independent clinical tissue samples, or blood-based measurements. Moreover, the source datasets primarily distinguish established cases from controls and do not provide longitudinal outcomes. The nomogram outputs therefore represent probabilities of case classification within the analyzed datasets rather than predictions of future disease risk. These models should be considered theoretical and hypothesis-generating. Before any clinical application, they require validation in independent, adequately powered cohorts with standardized diagnostic criteria, accessible clinical specimens, prespecified assay procedures, and comparison with established clinical predictors.

The shared inflammatory signatures identified in this study should be interpreted within the multifactorial pathogenesis of IDD and OP. In IDD, inflammatory signaling interacts with mechanical stress, impaired nutrient transport, oxidative damage, cellular senescence, and dysregulated matrix metabolism. In OP, immune signaling interacts with estrogen deficiency, aging, oxidative stress, genetic susceptibility, and altered osteoblast-osteoclast coupling. Therefore, the candidate genes identified here are unlikely to act as isolated disease determinants. They may instead reflect components of broader regulatory networks connecting immune activity with extracellular matrix and bone homeostasis. The potential biological relationship between IDD and OP may involve both systemic and local mechanisms. Systemic inflammaging and senescence-associated mediators could influence disc cells, osteoblasts, osteoclasts, and immune cells concurrently. Locally, changes in vertebral trabecular structure and cartilaginous endplate remodeling may affect mechanical loading and nutrient diffusion within the disc. However, the direction of the clinical association remains unresolved, and the present transcriptomic analyses cannot determine whether osteoporosis promotes disc degeneration, disc degeneration affects vertebral remodeling, or both arise partly from shared age-related processes.

A major limitation is that the cellular experiments evaluated only expression responses to TNF-α. Although NCF2 and CRH showed reproducible changes in HNPCs and HOBs, no gene-specific knockdown, overexpression, or rescue experiments were performed. Consequently, the present data cannot determine whether either gene directly regulates extracellular matrix degradation, apoptosis, oxidative stress, osteogenic differentiation, or mineralization. The immortalized cell lines and single-cytokine stimulation model also cannot reproduce the cellular heterogeneity, mechanical environment, aging-related changes, and systemic interactions involved in IDD and OP. Therefore, the cellular findings should be interpreted as preliminary expression-level support rather than functional or mechanistic validation. Targeted gene-perturbation studies in primary patient-derived cells, three-dimensional or organ-culture systems, and *in vivo* models will be required to establish causality. Several limitations should be considered when interpreting the present findings. First, the transcriptomic analyses were based on two small retrospective public datasets. GSE124272 contained whole-blood samples from patients with lumbar disc prolapse rather than intervertebral disc tissue from patients with histologically characterized IDD. Consequently, its transcriptional profile may reflect systemic responses associated with lumbar disc disease rather than molecular changes occurring directly within the degenerating disc. GSE35956 contained bone marrow-derived mesenchymal stromal cells from five elderly patients with primary osteoporosis and five younger non-osteoporotic donors. Differences in age, underlying indications for surgery, and other unmeasured clinical characteristics may therefore confound the observed OP-associated expression patterns. In addition, the datasets differed in tissue source and microarray platform. Although they were analyzed separately, these differences limit direct biological comparison and may affect the identification of shared genes. Second, the small sample sizes increase the risk of unstable differential-expression estimates, biomarker-selection variability, and model overfitting. Nested cross-validation reduced information leakage and provided a more conservative estimate of model performance, but it cannot replace validation in an independent cohort. The reported AUC values should therefore be interpreted as internal estimates rather than evidence of generalizable diagnostic accuracy. Similarly, the nomograms were derived from retrospective case-control transcriptomic data and represent theoretical classification models. They do not predict prospective disease risk and are not ready for clinical diagnosis or decision-making. Third, the experimental assessment was limited to three independently conducted experiments using immortalized HNPC and HOB cell lines. These experiments used the same cell lines and therefore did not capture biological variation among independent donors. Cellular immortalization may also alter gene-expression patterns and responses to inflammatory stimulation. Moreover, TNF-α treatment represents only one component of the complex pathological environments of IDD and OP. The experiments demonstrated inflammation-associated changes in NCF2, CRH, and BTK expression but did not include gene-specific knockdown or overexpression. They consequently do not establish whether these molecules directly regulate extracellular matrix degradation, apoptosis, mineralization, or osteogenic differentiation. Finally, the network pharmacology and molecular docking analyses were computational and hypothesis-generating. Database-derived compound-target associations may reflect network connectivity without indicating disease-specific relevance or therapeutic suitability. Docking scores estimate structural compatibility under computational assumptions but do not demonstrate physical binding, target engagement, biological activity, efficacy, pharmacokinetic suitability, or safety. The prioritized biomarkers and compound-target associations require confirmation using primary cells from multiple donors, patient-derived tissues, functional loss- and gain-of-function experiments, direct binding or target-engagement assays, appropriate animal models, and independent clinical cohorts. Accordingly, the present findings should be regarded as a foundation for further investigation rather than definitive mechanistic or clinical evidence.

## Conclusions

This study integrated transcriptomic profiling, bioinformatics, machine learning, network analysis, molecular docking, and preliminary *in vitro* experiments to investigate molecular features potentially linking intervertebral disc degeneration (IDD) and osteoporosis (OP). Five candidate biomarkers BTK, NCF2, CRH, FCGR3A, and SERPINA3 were prioritized through the analytical workflow. Functional analyses indicated that immune and inflammatory regulation, Fc receptor-related signaling, TNF/NF-κB-associated pathways, osteoclast differentiation, and extracellular matrix remodeling may contribute to the molecular relationship between IDD and OP.

TNF-α stimulation was associated with increased NCF2 and decreased CRH expression in immortalized HNPCs and HOBs, indicating that these genes are responsive to a defined inflammatory stimulus. However, these expression-level observations do not establish their causal or functional involvement in IDD- or OP-related cellular phenotypes. The identified genes should therefore be regarded as hypothesis-generating candidates rather than functionally validated biomarkers or therapeutic targets. Further studies incorporating gene-specific perturbation, primary patient-derived cells, independent clinical cohorts, and *in vivo* models are required to determine their mechanistic and translational relevance.

Taken together, the findings provide an exploratory framework for understanding inflammation-related molecular connections between IDD and OP and identify candidates for further investigation. The proposed biomarkers, nomograms, and compound-target interactions should be regarded as hypothesis-generating rather than clinically validated diagnostic or therapeutic tools. Independent clinical cohorts, primary cells or patient-derived models, gene-specific functional experiments, and *in vivo* studies are required to confirm the reproducibility, biological roles, and translational relevance of these findings.

## Data Availability

The original contributions presented in the study are included in the article/supplementary material. Further inquiries can be directed to the corresponding author.
